# A qualitative study exploring the needs related to the health system in women with experience of pregnancy termination due to fetal anomalies in Iran

**DOI:** 10.1186/s12884-020-03274-3

**Published:** 2020-09-29

**Authors:** Bahareh Kamranpour, Mahnaz Noroozi, Masoud Bahrami

**Affiliations:** 1 Department of Midwifery, College of Nursing and Midwifery, Rasht Branch, Islamic Azad University, Rasht, Iran; 2 Department of Midwifery and Reproductive Health, School of Nursing and Midwifery, Isfahan University of Medical Sciences, Isfahan, Iran; 3 Department of Adult Health Nursing, School of Nursing and Midwifery, Isfahan University of Medical Sciences, Isfahan, Iran

**Keywords:** Health system, Pregnancy termination, Fetal anomaly, Qualitative study, Iran

## Abstract

**Background:**

In order to provide appropriate and adequate care to women who have experienced termination of pregnancy due to fetal anomalies, the health needs of this group should be assessed. Considering the lack of information about the care and services required by these women in Iran, this study was conducted with the aim of exploring the needs related to the health system in women with experience of pregnancy termination due to fetal anomalies.

**Methods:**

This study was conducted with a qualitative approach. 40 participants were selected through purposive sampling, and the data were collected through in-depth semi structured interviews and field notes, and analyzed using conventional qualitative content analysis.

**Results:**

After analyzing the texts, the needs related to the health system in women with experience of pregnancy termination due to fetal anomalies were categorized in the three main categories: “efficient treatment team”, “optimal organizational structure in providing services” and “financial support for families”.

**Conclusions:**

The findings of the present study by exploring and highlighting the needs related to the health system in different dimensions in women with experience of pregnancy termination due to fetal anomalies can be helpful for designing and providing basic and comprehensive care programs.

## Background

Since birth of a healthy child is a part of feminine identity of a woman, pregnancy leading to fetal anomalies is a more destructive experience than any other type of loss [1]. Diagnosis of fetal anomalies is an unexpected event [2] and causes severe emotional, psychological and physical harm to women [3, 4]. Approximately 2–3% of pregnancies will be affected by congenital anomalies [5]. According to studies, most pregnant women with fetal anomalies choose to terminate their pregnancies [6]. In Iran, the prevalence rate of congenital anomalies is 2.6% and almost one in three pregnancies prenatally diagnosed with birth defects is now legally terminated [7]. The active role of women in termination of the fetal life is a unique issue that distinguishes the subsequent grief process from the grief caused by abortion or intra uterine fetal death (IUFD) [8–10]. Also, this event can cause many challenges for parents in terms of continuing pregnancy without intervention or experimental fetal treatment in specific cases [11, 12]. The need to make quick decisions about the status of pregnancy and termination of fetal life, given the current laws, the lack of palliative care, and the fear of social judgment, all cause these individuals to be in indescribable conflict with others. This complicates the process of caring for these people [5].

The stigma of having an abnormal fetus makes it difficult for women to share their experiences with others, and their grief becomes more complicated and severe [9, 13]. The results of studies indicate that these women do not receive enough support, and they cannot even mourn for their fetus [14]. As emotional support from family and others is lost over time, this lack of support leads to a need for more care resources that were not anticipated at the end of the pregnancy [15]. Undoubtedly, the experiences of these women in receiving care in the health system are effective in alleviating their worries and adapting to this difficult experience [16]. It is believed that due to the disgraceful social nature of abortion and the decision to terminate a pregnancy, the voices of women experiencing this event are not heard [17]. The results of Hutti^,^s study in women after fetal loss showed that caregivers need to plan purposeful, effective, and evidence-based interventions to improve the symptoms of bereaved couples [18].

There is little research on the proper and desirable care of this group of women and their families, and it is not yet clear whether the existing care and services are fully tailored to their needs [19–23]. Therefore, the present qualitative study was conducted with the aim of exploring the needs related to the health system in women with experience of pregnancy termination due to fetal anomalies.

## Methods

### Study design

This qualitative research is a part of an expanded mixed methods study which conducted between October 2017 and April 2018 and used a content analysis approach.

### Settings, sample and recruitment

In the present study, participants were women with experience of pregnancy termination due to fetal anomalies (*n* = 25), their husbands (*n* = 2), and healthcare providers who had experiences in caring or treating these women (*n* = 13) in Rasht city, Iran. These participants were selected through purposive sampling. The inclusion criteria were absence of any psychological disease (because of these diseases can cause changes in thinking and concentrating and this can affect the responses of the participants), willingness to participate in the study, providing informed consent, having the ability to understand questions, and a maximum of 1 year has passed since the termination of pregnancy. Eligible participants were accessed through pre-natal clinics, the offices of obstetricians, forensic medicine specialists and midwives and the midwifery wards of hospitals. They were recruited directly or called by phone. The first author (BK) did not have any role or relationship with the centers or participants. The demographic characteristics of the participants are presented in Table 1.

**Table 1:** Demographic characteristics of women with pregnancy termination due to fetal anomalies, their spouses and healthcare providers

Characteristic		Number
**Women and their spouses**	**Age (years)**	22–38
	**Education level**	Middle school (3), Diploma (8), Associate degree (2), BS (12), MS and Ph.D. (2)
	**Job status**	Employee (9), Housewife (7), Service (10), Freelance (1)
	**The time elapsed since pregnancy termination**	Less than 6 months (24), 6 months to one year (3)
	**Number of children**	No children (14), 1 to 2 children (13)
**Healthcare providers**	**Age (years)**	30–60
	**Work experience (years)**	1–30

### Data collection

Data collection methods included in-depth semi-structured individual interviews and field notes. The first author (BK) conducted the interviews and field notes. She had 12 years working experience in midwifery and was Ph.D. candidate in reproductive health. Other authors have previous interviewing experience and qualitative paper/report writing. Interviews started with the women with experience of pregnancy termination due to fetal anomalies using general questions including “Please explain how you felt when you found out about your fetal anomalies and that your pregnancy should be terminated? What needs in terms of providing care and services have you felt since then? Please explain about it?” Interviews began with spouses of women using a general question including “What did your spouse need in terms of providing care and services from diagnosis of fetal anomaly to the termination of her pregnancy? Please explain about it? Interviews began with healthcare providers using the general question including “In your opinion, what are the needs of women with experience of pregnancy termination due to fetal anomalies in terms of providing care and services in the health system? Please explain about it?” (See Additional files 1, 2 and 3 for copies of the topic guides). Then the open and interpretive responses of the participants guided the interview process. In this study, 40 interviews (45 to 60 min) were conducted in the participants’ desired locations and times. Interviews continued until data saturation occurred.

### Data analysis

In the present study each interview was recorded using an mp4 player. Data analysis was manually performed and no software was used for this purpose. Conventional qualitative content analysis was used in order to analyze the data. The interviews were transcribed verbatim by the first author (BK). The interviews were reviewed frequently to gain a full understanding of them. Then the sentences and phrases were coded and the same codes were merged in an inductive manner and those who had a similar concept fell into one category and formed sub-categories. Then, by comparing the sub-categories with each other, the categories which were conceptually similar were placed in a main category.

### Rigor and trustworthiness

In the present study combining several methods of data collection such as individual interviews, field notes and selecting participants with maximum variation strategy (in terms of age, educational level, occupation, number of children and time elapsed since pregnancy termination) were used to validate the data. In order to confirm the validity of the obtained contents, coded interviews were reviewed with four participating women with experience of pregnancy termination due to fetal anomalies in other sessions (member checking). Also, the opinions of four experts were used to match and ensure the consistency of the data with the participants’ statements. In the present study, in order to increase transferability, the findings of the study were presented to three women with similar characteristics of participating women who did not participate in the study to judge the similarity of the data to their experiences. This was done by obtaining the consent of the participating women and maintaining anonymity and confidentiality.

### Ethical considerations

Certificate of research was obtained from the ethics committee of Isfahan University of Medical Sciences (approved code: IR.MUI.Rec.1396.30206) and informed consent, anonymity, confidentiality of information and the right to withdraw at any time were observed. Also, the reasons for the study were explained prior to each individual interview.

## Results

After analyzing the data, 42 codes, nine sub-categories and three main categories were obtained. Three main categories include: efficient treatment team, optimal organizational structure in providing services and financial support for families (Table 2).

**Table 2:** Codes, sub-categories and main categories extracted from the data analysis

Code	Sub-category	Main Category
*Lack of empathy and support from caregivers * Lack of promising expression and behavior from caregivers * Lack of sufficient opportunity for the conversation with the doctor	Understanding the women’s condition by the treatment team	Efficient treatment team
* Lack of optimal and high quality care * Lack of expertise in service delivery *Lack of professional ethics and respect for patients’ rights	Professional competence of the treatment team in providing care	
* Lack of health insurance *High share of people in paying diagnostic and screening costs * Inadequate coverage of most services by health insurance	Adequate coverage of insurance services for diagnostic and follow-up costs	Financial support for families
*High costsof diagnostic and screening tests * High counseling services costs *Failure to follow the course of treatment due to financial problems	Financial ability of families to pay diagnostic, treatment and consulting costs	
*Incompatibility of the number of caregivers with the number of patients *Long waits in wards to receive services, due to lack of human resources	Existence of the necessary and sufficient human resources	Optimal organizational structure in providing services
*Inappropriate physical environment in the maternity ward *Lack of suitable space for communication with family members	Favorable physical environment in the maternity ward	
* Lack of attention to the psychological problems of clients in the course of treatment * Need to provide counseling services in the hospital (before discharge) * The need for a psychologist in health care centers	Existence of organized counseling and supportive services in health centers	
*Lack of amenities such as TV, refrigerator and wardrobe for clients and companions * Lack of sofa bed for the companions * Inadequate quality of food and lack of access to restaurants or coffee shops *Lack of independent toilets and bathrooms for each room * Existence of old and non-standard hospital beds in the wards	Existence of appropriate welfare facilities for women and their companions	
*Distribution of diagnostic, treatment and forensic medicine centers in different places * Spending a lot of energy and time to access diagnostic and screening centers * Spending a lot of energy and time to go to the forensic medicine center to get a pregnancy termination license	Accumulation of services in one place	

### Efficient treatment team

According to the participants, in order to meet the needs of women with experience of pregnancy termination due to fetal anomalies, it is necessary to provide them with comprehensive care by efficient treatment team. This main category includes two sub- categories.

#### Understanding the women’s condition by the treatment team

According to the participating women, one of their most important needs during and after pregnancy termination is the need for understanding their condition by the members of the treatment team. They asked for behavior with empathy and kindness, hopeful expression, and attention to their concerns about the pregnancy termination procedure.

*"Healthcare providers are very frustrated in some shifts; they don't care about our situation at all. … When I walked down the hall, they said: 'why are you walking? The ward is crowded, go into the room.'" (A woman)*

#### Professional competence of the treatment team in providing care

A number of participating women complained about the lack of necessary skills in the members of the treatment team and their uncertainty of receiving effective treatment. They believed that healthcare providers should have the necessary abilities for their services and provide cares within the professional standards. According to the participating women, healthcare providers should have up-to-date knowledge and spend sufficient time to educate and answer women’s questions.

*" … They (healthcare providers) carry out the doctor's instructions without explaining. They don't say what they are doing at all!!! Their behavior is not right at all!!! They don't respect anyone."(A woman)*

### Optimal organizational structure in providing services

The statements of the participants included cases that resolving them required the creation of favorable organizational structure to provide high-quality services, including: existence of the necessary and sufficient human resources; favorable physical environment in the maternity ward; accumulation of services in one place; existence of appropriate welfare facilities for women and their companions; and existence of organized counseling and supportive services in health centers. This main category includes five sub- categories.

#### Existence of the necessary and sufficient human resources

Most of the participating women, especially in educational hospitals, stated that the number of healthcare providers is not enough and this prevents the proper services to patients who need special care due to special conditions.

*" … They (healthcare providers) didn't pay attention at all…. There were a lot of patients, but there were few staff. They couldn't take care everyone!" (A woman)*

#### Favorable physical environment in the maternity ward

Participating women emphasized the existence of an environment where the patient’s privacy is maintained and the possibility of communication between patient and her family members is obtained during the hospital stay.

*"The maternity ward was very awkward. The rooms were very narrow and really difficult for the patients to pass by." (A woman)*

Participating midwives believed that the structure and physical environment of the maternity and inpatient wards affect the quality of cares and services as well as women’s satisfaction.

*“ … The environment of the maternity ward is very crowded especially in the morning shifts, which can affect the quality of care and patient satisfaction.” (A midwife)*

#### Accumulation of services in one place

Participating women narrated that they had to go to different centers to receive approval of fetal anomalies and also obtain a certificate for pregnancy termination from forensic medicine center and therefore a lot of their money, energy and time is wasted. Also, they complained about the length and complexity of the process of receiving license for pregnancy termination from forensic medicine center. They found it very painful in their critical situation.

*“I wish we could get the letter (abortion license) from somewhere else rather than forensic medicine center. This was very painful!!! … . I wish we could get the letter in the hospital.”(A woman).*

#### Existence of appropriate welfare facilities for women and their companions

According to the participating women, the lack of adequate welfare facilities for them and their companions in hospitals, especially in governmental centers makes them more annoyed. They pointed to the lack of a direct telephone line, TV, refrigerator, cupboard, independent toilet and bathroom, a sofa bed and a daily food menu for their companions in each room.

*“The facilities at the hospital were not good. … They don't provide a comfortable place with my companion. ..." (A woman)*

#### Existence of organized counseling and supportive services in health centers

Participating women narrated that they need a lot of psychological support. This makes it easier for them to accept this event.

*"I wanted my doctor to support me more and to really prepare me psychologically. I really had a great hard time after the termination of my pregnancy.” (A woman)*

According to participating healthcare providers, the focus of the members of the treatment team in most cases is on providing physical care and very poor psychological support is provided. They believed that the members of the treatment team should have the necessary skills in providing supportive care to make it easier for the woman to accept this event and prevent her from suffering psychological trauma. According to participating healthcare providers, policy-making and providing a proper platform in health care centers to provide organized psychological support to women is a necessity for most women. Also, they narrated that psychiatric counseling should be provided to these women before entering the maternity ward, so that they can receive the necessary care, identify psychological problems, and refer them to counseling centers.

*“The period of stress and psychological damage begins from the moment that these women hear the news of fetal anomalies. Therefore, the medical staff must have the necessary skills to provide supportive care for them.” (A psychologist)*

### Financial support for families

Participants in present study considered the need for financial support for families, including adequate coverage of insurance services and having the financial ability to pay the costs. This main category includes 2 sub-categories.

#### Adequate coverage of insurance services for diagnostic and follow-up costs

According to the participating women and their spouses, the high cost of screening and diagnostic tests led to a number of them are refusing to receive the necessary tests to prepare for next pregnancy.

*“I want to get pregnant again, but my doctor said: you should have the genetic tests and the tests are expensive. So, my husband and I decided to wait until we see how things turn out.” (A woman)*

Participating healthcare providers considered sufficient insurance coverage in services (including counseling services), people’s small share of payments and timely payment by insurance companies necessary.

*" … Due to the high cost of follow-up and diagnostic tests, these costs must be covered by insurance.” (An obstetrician)*

#### Financial ability of families to pay diagnostic, treatment and consulting costs

Participating women and their spouses narrated that since services such as genetic counseling, psychological counseling, as well as advanced screening and diagnostic tests needs high costs and multiple referrals; many families can’t afford the costs.

*"When I got the abortion license, I didn't want to go to a governmental center. But we don't have enough money!!! So, we came to the governmental maternity hospital."(A woman)*

Participating healthcare providers believed that financial support from organizations as an important step in supporting families with financial problems.

*“Although many women need counseling services, because of the inability to pay, they refuse to do it. Therefore, these families should be supported.*” *(A reproductive health specialist)*

## Discussion

This study was conducted with the aim at exploring the needs related to the health system in women with experience of pregnancy termination due to fetal anomalies. The results of the present study showed that efficient treatment team, optimal organizational structure in providing services and financial support for families are the most important needs related to the health system in these women. According to the majority of participants in the present study, having mastery, respectful behavior, and having a satisfactory relationship are all factors that lead to women’s confidence in the professional competence of the treatment team. According to them, healthcare providers have somewhat forgotten other aspects of their professional care, such as empathy with patients, due to increased workload. The results of some studies also indicated that proper communication with patients will lead to the improvement of therapeutic outcomes [3, 24]. In this regard, the results of other studies showed that proper communication between healthcare providers, skills in performing care and providing training to clients, more than other aspects affect patient satisfaction [25, 26]. Therefore, it is necessary for healthcare providers to have the necessary skills to improve their competencies with the right behavior, work conscience and responsibility. Most patients demand good quality services due to the increase in the level of health awareness and the increase in the cost of health services [25, 27]. Improving the quality of services in performing care is the most important factor that can accelerate the recovery of patients. It is believed that in most cases the goal of the members of the treatment team is only to provide physical care to the client and, the low of human resources and the use of inexperienced staff lead to the decrease in the quality of care and client dissatisfaction [28].

The narrations of the participants indicated that most women were afraid of pregnancy termination procedure. This suggests that providing pre-procedure information is insufficient or ineffective [29]. During an abortion, a woman is psychologically sensitive and vulnerable, creating a calm environment, respecting her, starting an examination with her permission, and observing her privacy can provide her with more peace of mind [30, 31]. In addition to the medical and educational aspects, focusing on women’s emotional needs and concerns is important. Therefore, the supportive care team will be necessary at this time.

Most of the participants in the present study believed that the number of human resources was insufficient and this prevented them from providing proper and timely services. They also complained about the inappropriate physical environment of the inpatient wards. Lotto et al. showed that reducing the number of professional caregivers has the opposite effect on patient care quality ^5]^. Many participants in Pitt et al.’s study also complained about the poor physical environment of inpatient wards [19].

The results showed that since women with pregnancy termination due to fetal anomalies need special care including various consultations, it is not possible to protect the patient’s privacy. The results of Choi & Busch’s study showed that the patient rights were more observed in private rooms, which led to greater patient satisfaction with the medical center [32]. According to the results of the present study, in the process of pregnancy termination, the psychological issues of the woman and her family are not considered and only the procedure of her pregnancy termination is considered. Therefore, a special trustee should be considered for psychological support in medical and health centers. The results along with the findings of several other studies, showed that providing expert advice in these women requires a specialized team. Such women will need to provide counseling on a regular basis [33, 34]. So, it is necessary to create a team consisting of specialists in fields such as obstetrics, pediatrics, genetics, medical ethics, religious counselor, psychiatrist or clinical psychologist and lawyer [35, 36]. Obviously, this will facilitate a conscious decision-making process [37].

Based on the results, the lack of allocation of welfare facilities to the women with pregnancy termination due to fetal anomalies and their companions in medical settings (especially in governmental centers) annoyed them. In this regard, the results of Pitt and Howard’s studies also emphasized the need to pay attention to the welfare facilities required in the inpatient wards and provide family-centered care [19, 38]. Also, the accumulation of services in one place was another requirement presented by the participants in the present study. In this regard, the results of Asplin and Fisher^,^ s studies showed that the dispersion of diagnostic, treatment and service centers causes many problems and wastes a lot of money, energy and time [16, 39]. Since the insurance status of clients determines easier access to the required services [40], a number of participating women in the present study were dissatisfied with the heavy costs of diagnostic tests, the required counseling (including genetic counseling and psychological counseling), post-abortion examinations, and insufficient insurance coverage. In this regard, the results obtained by Cacciatore et al. also showed that parents were unable to perform genetic counseling, due to poor economic conditions and refused to do so [41].

### Recommendations

According to the results, existence of a skilled and supportive care and treatment team; paying attention to the welfare facilities required in the inpatient wards; reducing the dispersion of diagnostic, treatment and service centers; providing psychological support in medical and health centers; and adequate insurance coverage for women with experience of pregnancy termination due to fetal anomalies is essential.

### Study limitations

Since the details of events are forgotten as time goes on, therefore, women that it has been more than 1 year of their pregnancy termination due to fetal anomalies were not included in the present study. Considering the existence of studies that have reported the continuation of the consequences for more than 1 year, limiting the samples to a maximum of 1 year can be one of the limitations of the present study.

## Conclusion

This study showed the needs related to the health system in women with experience of pregnancy termination due to fetal anomalies in different dimensions. It seems that exploring and highlighting these needs can pave the way for healthcare providers who play an important role in meeting the needs of these people. Also, health planners and authorities can identify and address the problems in the health system by being aware of these needs.

## Supplementary information

**Additional file 1.**

**Additional file 2.**

**Additional file 3.**

## Data Availability

The datasets generated and/or analysed during the current research are not publicly available as individual privacy could be compromised but are available from the corresponding author on reasonable request.

## Abbreviation

IUFD: Intra Uterine Fetal Death
