# Supplementary material for: A qualitative study exploring the needs related to the health system in women with experience of pregnancy termination due to fetal anomalies in Iran
Source: BMC Pregnancy Childbirth. 2020 Sep 29;20:573. doi: 10.1186/s12884-020-03274-3 (PMC7526095; doi:10.1186/s12884-020-03274-3)
Supplement: Supplementary file 1 — Additional file 1. [file 12884_2020_3274_MOESM1_ESM.docx]

**Additional files**

**Additional file 1:** Interview guide during the face-to-face interviews with women with experience of pregnancy termination due to fetal anomalies for the study conducted to explore the needs related to the health system from the perspective of women, their spouses and healthcare providers in Rasht Town, Iran, 2017-2018 (See methods section for further description).

**Introduction:** *Aim, to create appropriate atmosphere*

- Name of the interviewer and affiliation
- Purpose of the study
- Consent to take part in the study
- Confidentiality, explain how the data will be used
- Interview will last approximately 30-60 minutes
- Audio recorded to ensure interviewer can fully engage in the interview

**Warm up questions:** *Aim\ make participants comfortable*

1. Please introduce yourself?

2. How old are you?

3. What is your education level?

4. What is your job?

5. How many pregnancies have you had?

6. How many children do you have?

7. How long has it been since your pregnancy terminated?

8. What was your gestational age at the time of the termination of pregnancy?

**Interview guide questions in individual interviews with women with experience of pregnancy termination due to fetal anomalies**

1. Please explain how you felt when you found out about your fetal anomalies and that your pregnancy should be terminated?

2. What needs in terms of providing care and services have you felt since then? Please explain about it?

3. How could healthcare providers (midwives, nurses, obstetricians, forensic medicine specialists, reproductive health specialists and psychologists) help you? Please explain?

4. What would you like healthcare providers to do for you? Please explain?

5. How would you like healthcare providers to help you after your discharge from the hospital? Please explain?

6. In general, what do you expect from the health system to improve your situation?
