# Supplementary material for: A qualitative study exploring the needs related to the health system in women with experience of pregnancy termination due to fetal anomalies in Iran
Source: BMC Pregnancy Childbirth. 2020 Sep 29;20:573. doi: 10.1186/s12884-020-03274-3 (PMC7526095; doi:10.1186/s12884-020-03274-3)
Supplement: Supplementary file 2 — Additional file 2. [file 12884_2020_3274_MOESM2_ESM.docx]

**Additional file 2:** Interview guide during the face-to-face interviews with spouses of women with experience of pregnancy termination due to fetal anomalies for the study conducted to explore the needs related to the health system from the perspective of women, their spouses and healthcare providers in Rasht Town, Iran, 2017-2018 (See methods section for further description).

**Introduction:** *Aim, to create appropriate atmosphere*

- Name of the interviewer and affiliation
- Purpose of the study
- Consent to take part in the study
- Confidentiality, explain how the data will be used
- Interview will last approximately 30-60 minutes
- Audio recorded to ensure interviewer can fully engage in the interview

**Warm up questions:** *Aim\ make participants comfortable*

1. Please introduce yourself?

2. How old are you?

3. What is your education level?

4. What is your job?

5. How many children do you have?

6. How long has it been since your spouse’s pregnancy termination?

**Interview guide questions in individual interviews with** **spouses of women with experience of pregnancy termination due to fetal anomalies**

1. What did your spouse need in terms of providing care and services from the time of the diagnosis of fetal anomaly to the termination of her pregnancy and afterward? Please explain about it?

2. What expectations did your spouse have from the healthcare providers (midwives, nurses, obstetricians, forensic medicine specialists, reproductive health specialists and psychologists) after termination of her pregnancy? Please explain?

3. In your opinion, how can healthcare providers (midwives, nurses, obstetricians, forensic medicine specialists, reproductive health specialists and psychologists) help women with experience of pregnancy termination due to fetal anomalies? Please explain?

4. In general, what do you expect from the health system to improve your spouse’s situation?
