# Supplementary material for: A qualitative study exploring the needs related to the health system in women with experience of pregnancy termination due to fetal anomalies in Iran
Source: BMC Pregnancy Childbirth. 2020 Sep 29;20:573. doi: 10.1186/s12884-020-03274-3 (PMC7526095; doi:10.1186/s12884-020-03274-3)
Supplement: Supplementary file 3 — Additional file 3. [file 12884_2020_3274_MOESM3_ESM.docx]

**Additional file 3:** Interview guide during the face-to-face interviews with health care providers (midwives, nurses, obstetricians, forensic medicine specialists, reproductive health specialists and psychologists) for the study conducted to explore the needs related to the health system from the perspective of women, their spouses and healthcare providers in Rasht Town, Iran, 2017-2018 (See methods section for further description).

**Introduction:** *Aim, to create appropriate atmosphere*

- Name of the interviewer and affiliation
- Purpose of the study
- Consent to take part in the study
- Confidentiality, explain how the data will be used
- Interview will last approximately 30-60 minutes
- Audio recorded to ensure interviewer can fully engage in the interview

**Warm up questions:** *Aim\ make participants comfortable*

1. Please introduce yourself?

2. How old are you?

3. What is your education level?

4. What is your job?

5. What is your work experience?

**Interview guide questions in individual interviews with healthcare providers**

1. In your opinion, what are the needs of women with experience of pregnancy termination due to fetal anomalies in terms of providing care and services in the health system? Please explain about it?

2. What can healthcare providers (midwives, nurses, obstetricians, forensic medicine specialists, reproductive health specialists and psychologists) do to meet the needs of these women from the time of the diagnosis of fetal anomalies to termination of pregnancy and afterward?

3. In your opinion, what should health policymakers do to improve the status of women with experience of pregnancy termination due to fetal anomalies?
